# Identification of Mitochondrial-related Characteristic Biomarkers in Osteosarcoma using Bioinformatics and Machine Learning

**DOI:** 10.2174/0113862073350964241203080017

**Published:** 2025-02-25

**Authors:** Jingyi Hou, Yu Zhang, Ning Yang, Bin Chen, Chengbing Chang, Haipeng Gu, Yanqi Liu, Naiqiang Zhu

**Affiliations:** 1 Department of Minimally Invasive Spinal Surgery, The Affiliated Hospital of Chengde Medical University, Chengde 067000, China;; 2 Chengde Medical University, Chengde, 067000, China;; 3 Central Laboratory, The Affiliated Hospital of Chengde Medical University, Chengde, 067000, China;; 4 Hebei Key Laboratory of Panvascular Diseases, The Affiliated Hospital of Chengde Medical University, Chengde, 067000, China

**Keywords:** Osteosarcoma, mitochondrial-related differentially expressed genes, machine learning, UCP2, PRDX4, immune cell infiltration

## Abstract

**Background/aims:**

Osteosarcoma (OS), A malignant tumor originating in bone or cartilage, primarily affects children and adolescents. Notably, substantial alterations in mitochondrial energy metabolism have been observed in OS; however, the specific contribution of Mitochondrial-Related Genes (MRGs) to OS pathogenesis and prognosis remains unclear. Herein, we identified novel diagnostic biomarkers associated with mitochondrial-related processes in OS *via* comprehensive bioinformatics analysis.

**Methods:**

OS mRNA expression profiles were retrieved from GSE16088 and GSE19276 databases. Mitochondrial-related Differentially Expressed Genes (MitoDEGs) were identified by integrating differentially expressed analysis with mitochondrial-localized genes. A protein-protein interaction network was constructed, and machine learning algorithms (LASSO regression analysis and SVM-RFE) identified characteristic MitoDEGs. Subsequently, immune cell infiltration, microenvironment analysis, and single-cell RNA sequencing (scRNA-seq) analyzed differences in characteristic MitoDEGs, and RT-PCR was used for *in vitro* verification of characteristic MitoDEGs.

**Results:**

MitoDEGs in OS were significantly enriched in the pathways associated with mitochondrial function and immune regulation. Two MitoDEGs, UCP2 and PRDX4, were identified *via* LASSO and SVM-RFE. Correlation analysis demonstrated a close association between UCP2 and PRDX4 expression levels and immune cell infiltration, particularly in CD8+ T and native CD4+ T cells, as observed in both immune cell and scRNA-seq analyses. Furthermore, RT-PCR confirmed the expression levels of UCP and PRDX4 at the cellular level, which was consistent with the bioinformatics results.

**Conclusion:**

This study identified UCP2 and PRDX4 as characteristic MitoDEGs and potential diagnostic biomarkers for OS using machine learning algorithms. These findings provide novel insights into the clinical applications of these biomarkers for OS diagnosis.

## 1 INTRODUCTION

Osteosarcoma (OS) is a malignant tumor of the bone or cartilage that primarily affects children and adolescents, making it the third most common malignancy in this demographic [1, 2]. The Clinical manifestations of OS include local pain, swelling, functional impairment, and pathological fractures, often accompanied by metastases to the lungs, bones, and other sites, considerably impacting the patients' quality of life and psychological well-being [3, 4]. The development and progression of OS involve various genetic and environmental factors such as chromosomal abnormalities, growth factors, and cytokines; however, the precise mechanisms remain unclear. OS diagnosis currently relies primarily on radiology and pathology; however, owing to the high heterogeneity and diversity of OS, its classification and grading remain controversial and challenging.

Conventional diagnostic methods, which predominantly utilize imaging modalities such as Magnetic Resonance Imaging (MRI) and Computed Tomography (CT) scans alongside histopathological analysis, frequently encounter notable limitations. For instance, imaging may not reliably differentiate OS from other benign bone lesions, which can lead to potential misdiagnoses. Additionally, histological assessments are often subjective and can be significantly affected by tumor heterogeneity, which varies widely across different patient populations and disease stages. This heterogeneity contributes to substantial disparities in treatment efficacy, as conventional chemotherapy and surgical interventions show variable responses depending on tumor subtype and individual genetic backgrounds [3-6]. These challenges highlight the urgent need for innovative diagnostic tools capable of enabling more precise and personalized patient stratification.

Recent studies have increasingly focused on mitochondrial genetics as a promising pathway for biomarker discovery. Mitochondria function as cellular powerhouses and oxidize sugars, fatty acids, amino acids, and other substances to generate adenosine triphosphate (ATP) and other metabolic intermediates, thereby maintaining cellular function [7, 8]. Mitochondrial energy metabolism refers to a series of chemical and energy reactions within the mitochondria, crucially influencing energy production and consumption and impacting cellular processes such as proliferation, differentiation, apoptosis, autophagy, oxidative stress, inflammation, and immunity [9, 10]. Substantial alterations in mitochondrial energy metabolism have been observed in OS and are closely associated with the initiation of its progression. For example, in OS, alterations in gene expression and function accompany changes in both the quantity and structure of mitochondria, resulting in the disruption of mitochondrial energy metabolism. Furthermore, mitochondria can regulate the biological behavior and death pathways of OS cells *via* cellular metabolism [11, 12]. Additionally, unlike conventional biomarkers, mitochondrial genes may offer insights into tumor behavior that are specific not only to the tumor type but also to individual patient profiles. This specificity could enable more refined risk stratification and personalized treatment strategies, addressing the limitations of current diagnostic practices, which often rely on generalized approaches. The limitations of current diagnostic and therapeutic approaches for OS highlight the urgent need for novel biomarkers in clinical practice. Given that treatment outcomes remain inconsistent and often inadequate for many patients, integrating mitochondrial biomarkers could transform the diagnosis and management of this aggressive malignancy, ultimately improving patient outcomes and survival rates.

Recently, bioinformatics has facilitated the prediction of disease biomarkers and the analysis of their underlying molecular mechanisms. These developments offer improved conceptual frameworks and methodologies for subsequent clinical investigations [13, 14]. In this study, we aimed to identify novel biomarkers associated with mitochondrial-related processes in OS based on comprehensive bioinformatics analysis, offering a novel perspective for clinical immunotherapeutic interventions and therapeutic strategies for OS treatment.

## 2 MATERIALS AND METHODS

### 2.1 Data Collection and Processing

OS datasets were retrieved from the Gene Expression Omnibus (GEO) database (http://www.ncbi.nlm.nih.gov/geo) using the search term "osteosarcoma." Two datasets, GSE16088 and GSE19276, were selected for subsequent analyses. The GSE16088 dataset, associated with the GPL96 platform (Affymetrix Human Genome U133A Array) and deposited in 2009, comprises transcriptomic analyses of 14 OS samples and 3 samples from healthy individuals (Nor) [15]. Moreover, the GSE19276 dataset, utilizing the GPL6848 platform (Agilent-012391 Whole Human Genome Oligo Microarray G4112A) and deposited in 2009, consists of 28 samples, with 23 from OS specimens and 5 from healthy individuals [16]. The single-cell RNA sequencing (scRNA-seq) data for OS were obtained from GSE162454, which includes six patients with OS in the GEO database [17]. Additionally, a collection of 1,136 genes localized to the mitochondria (Supplementary Materials) was obtained from MitoCarta 3.0 (https://www.broadinstitute.org/mitocarta/) [18]. The following steps were performed on GSE16088 and GSE19276: (1) chip probes were mapped to the human genome using the Bioconductor package in R and (2) expression profiles of mitochondria-related genes were retained.

### 2.2 Differentially Expressed Gene (DEG) and Functional Enrichment Analyses

Microarray gene expression profiling data underwent standardization and correction, including gene name annotation and identification of DEGs, using the "limma" package within R (Version 4.34; https://www.r-project.org). A conservative criterion (|log2FC| >1, *p*-value <0.05) was used to detect DEGs between the Nor and OS samples in both GSE16088 and GSE19276 datasets. Subsequently, the expression patterns of DEGs were visually represented using volcano plots from the "ggplot2" package and heatmaps generated with the "ComplexHeatmap" package in R. Furthermore, Gene Ontology (GO) and Kyoto Encyclopedia of Genes and Genomes (KEGG) pathway enrichment analyses were conducted to elucidate distinct biological and functional characteristics using the "clusterProfiler" (Version 3.14.3) and "org.Hs.eg.db" packages in R [19]. The relevant functional map is considered to be significantly rich if it meets the threshold of *p*-value < 0.05, and visualization was conducted using the "ggplot2" package in R.

### 2.3 Identification of Mitochondrial-related DEGs (MitoDEGs)

MitoDEGs were identified by intersecting DEGs with 1,136 mitochondrial genes using Venn diagram analysis in both the GSE16088 and GSE19276 datasets. The overlapped MitoDEGs from both microarrays were subsequently visualized as a Heatmap using the “ggplot2” package in R. Functional enrichment analysis was performed using “clusterProfiler” and “org.Hs.eg.db” packages in R and the Metascape database (https://metascape.org/gp/index) [20].

### 2.4 Protein-protein Interaction (PPI) Network Construction of MitoDEGs

MitoDEGs were submitted to the STRING database (https://string-db.org/, Version 11.0, accessed on 18 December 2021) to construct a PPI network with a combined score threshold set at > 0.4. The network was visualized with Cytoscape (https://cytoscape.org/, Version 3.7.2). The CytoHubba (Version 1.0) plug-in was used to calculate the topological parameters of the nodes and interaction edges within the network using the degree algorithm. As previously reported, the degree algorithm effectively identifies important nodes in the network, with nodes having the highest degree score recognized as key targets [21].

### 2.5 Selection of Characteristic Genes Among MitoDEGs via using Machine Learning

Least absolute shrinkage and selection operator (LASSO) regression analysis and SVM-RFE algorithms [22] were employed to identify characteristic genes among the key MitoDEGs. LASSO analysis served as a dimensionality reduction technique, using a penalty parameter *via* the "glmnet" package. SVM-RFE analysis was implemented for classification and regression, and hyperparameters for all classifiers were selected, facilitated by the "Caret" and "e1071" packages in R. In this study, candidate genes associated with characteristic genes of MitoDEGs were selected using LASSO and SVM-RFE feature selection methods. To enhance model performance, we employed 10-fold cross-validation for training. Furthermore, expression levels of the selected genes were validated using the GSE16088 and GSE19276 datasets to evaluate their potential as candidate diagnostic biomarkers.

### 2.6 Immune Infiltration and Tumor Microenvironment (TME) Analysis

The “limma” package in R normalized the data from GSE16088 and GSE19276 to estimate the proportion of infiltrating immune cells. Thereafter, standardized gene expression data underwent CIBERSORT analysis using the LM22 signature and 1,000 permutations. The associations between potential diagnostic biomarkers and immune cells showing significant changes were analyzed using the Spearman correlation coefficient with the aid of the 'reshape2' and 'ggExtra' R packages [1]. In addition, ESTIMATES, Immune, Stromal, and TumorPurity scores for each sample were determined using the ESTIMATE algorithm implemented in the 'estimate' package.

### 2.7 Single-cell RNA Sequencing Analysis of Characteristic MitoDEGs

Data from the GEO and Array Express databases were aggregated using the Tumor Immune Single-Cell Hub (TISCH, http://tisch.comp-genomics.org/) to construct a comprehensive scRNA-seq atlas. TISCH facilitates comparative analyses across various patient cohorts, treatment regimens, response patterns, tissue origins, cellular phenotypes, and cancer subtypes, enabling the visualization of gene expression dynamics at both single-cell and cluster resolutions [23]. For this investigation, we used TISCH datasets from GSE162454 to elucidate the characteristic genes of MitoDEGs at the single-cell level.

### 2.8 Cell Culture and RT-qPCR Verification

Four OS cell lines (Saos2, MG63, HOS, and U2OS) along with osteoblasts (hFOB 1.19) were sourced from the Cell Bank of the Chinese Academy of Sciences (Beijing, China). Cells were maintained in DMEM (Gibco, USA) containing 1% penicillin-streptomycin and 10% fetal bovine serum (Gibco, USA) at 37°C with 5% CO₂. The culture medium was refreshed every 24–48 hours. Cells were passaged when confluence exceeded 80%, following trypsin-EDTA digestion (Beyotime, USA). Total RNA was extracted using the FastPure RNA Extraction Kit (TIANGEN, China). RNA concentration was determined using a Nanodrop spectrophotometer. cDNA synthesis was performed using a qPCR RT kit (TIANGEN), and SYBR qPCR Master Mix was used for mRNA expression measurements. The primer sequences used were as follows: UCP2 (F: 5’-AGCTTTGAAGA ACGGGACAC-3’, R: 3’-CTGCTACGTCCCAGGAGATG-5’) and PRDX4 (F:5’-TCTTTCAGATTTGACCCATCAG-3’, R: 3’-TCATCCACTGATCTACCCACAG-5’) and GAPDH (F: 5’-AATGGGCAGCCGTTAGGAAA-3’, R: 3’-GCCC AATAC GACCAAATCAGAG-5’). Gene expression levels were normalized to GAPDH and calculated using the 2^−ΔΔCt ^method.

### 2.9 Statistical Analyses

Bioinformatics statistical analyses were performed using the R software (version 4.1.3, https://www.r-project.org/). Group differences were evaluated *via* one-way analysis of variance (ANOVA) followed by Tukey's post-hoc test. Statistical analyses of cell experiment data were conducted in GraphPad Prism 9.0, with results expressed as mean ± SD. A *p*-value < 0.05 was considered statistically significant.

## 3 RESULTS

### 3.1 Workflow Analysis and Data Description

A simplified depiction of the workflow is presented in Fig. (**1**). First, we identified DEGs and MitoDEGs in GSE16088 and GSE19276, respectively. Thereafter, a PPI network of shared MitoDEGs was constructed, and characteristic genes among the key targets of MitoDEGs were identified using LASSO regression analysis and SVM-RFE machine learning algorithms. Immune cell infiltration, microenvironments, and scRNA-seq analyses were performed to determine the differences in selected characteristic genes of MitoDEGs. Furthermore, RT-PCR was used for *in vitro* verification of these characteristic genes at the cellular level.

### 3.2 Identification of DEGs in OS and Functional Enrichment Analysis

As shown in Figs. (**2A**, **B**), from GSE16088, 1578 and 1182 DEGs were identified. Among these, GSE16088 exhibited 810 downregulated and 768 upregulated DEGs. GO analysis suggested that the DEGs in the GSE16088 datasets were primarily enriched in cell proliferation, osteoblast differentiation in biological process terms. Moreover, molecular function terms highlighted associations with ATPase activity (Fig. **2C**). Furthermore, the most enriched KEGG pathways of the DEGs in the GSE16088 datasets were predominantly enriched in ECM-receptor interactions, cell cycle, osteoclast differentiation (Fig. **2D**). In contrast, the examination of the GSE19276 dataset, as detailed in Figs. (**2E**, **F**), uncovered a total of 1182 DEGs. The expression profile within this dataset is nearly equitable, with 602 DEGs showing a decrease in expression and 580 exhibiting an increase. GO analysis of these DEGs revealed a significant enrichment in cellular components such as the nucleoplasm, nucleus, cytosol, and extracellular exosome, indicating a potential involvement in a range of cellular processes and extracellular signaling in the cellular component terms. The molecular function category of the GO analysis further emphasized a strong association with the extracellular matrix's structural components, as depicted in Fig. (**2G**). Moreover, the KEGG pathway analysis of the DEGs in the GSE19276 dataset prominently featured enrichment in cytokine-cytokine receptor interaction pathways, as illustrated in Fig. (**2H**).

### 3.3 Identification of MitoDEGs and Functional Enrichment Analysis

Genes associated with mitochondria were obtained from the MitoCarta3.0 database. Those genes that overlapped with DEGs in the datasets were classified as MitoDEGs. As shown in Figs. (**3A**, **B**), 124 MitoDEGs were identified in GSE16088. GO functional enrichment analysis indicated that these MitoDEGs were mainly linked to processes related to mitochondrial gene expression, the mitochondrial matrix, inner and outer membranes, oxidoreductase activity (acting on NADPH), mitochondrial ribosomes, as well as NADP binding (Fig. **3C**). Additionally, KEGG pathway analysis suggested that the MitoDEGs in GSE16088 were primarily associated with tricarboxylic acid (TCA) cycle, 2-oxcarboxylic acid metabolism, oxidative phosphorylation, pyruvate metabolism (Fig. **3D**). As shown in Figs. (**3E**, **F**), 82 MitoDEGs were identified in GSE19276. GO functional enrichment analysis indicated that these MitoDEGs were mainly linked to processes related to NADP dehydrogenase activity, suggesting their roles in mitochondrial functions (Fig. **3G**). Moreover, KEGG pathway analysis suggested that the MitoDEGs in GSE19276 were primarily associated with carbon metabolism (Fig. **3H**).

### 3.4 PPI Network Construction of MitoDEGs

As shown in Fig. (**4A**), 24 MitoDEGs were identified *via* the intersection of DEGs from datasets GSE16088 and GSE19276 with genes cataloged in MitoCarta 3.0. The obtained MitoDEGs were subsequently analyzed within the STRING database to construct a PPI network using a combined score threshold >0.4. The resulting network was visualized using Cytoscape 3.7.2, with 21 nodes and 78 interaction edges (Fig. **4B**). The topological parameters of the network were computed using the degree algorithm within the CytoHubba plug-in to assess the significance of the nodes within the network. Notably, MRPL12, MRPS7, ECHS1, HSPD1, HSPA9, MTIF2, CS, MPRL11, MRPS16, PHB, SLIRP, TRAP1, TFB2M, ETFA, MRPS28, GOT2, FASN, TXNRD1, peroxiredoxin 4 (PRDX4), uncoupling protein 2 (UCP2), and DHRS2 exhibited high degrees of expression and were designated as pivotal genes among the MitoDEGs. Furthermore, we found that MitoDEGs were primarily enriched in GO terms associated with mitochondrial function, including processes such as mitochondrial organization, the TCA cycle, mitochondrial protein import, and mitochondrial gene expression (Fig. **4C**).

### 3.5 UCP2 and PRDX4 Screening Out Based on Machine Learning Algorithms

As shown in Figs. (**5A**, **B**), the LASSO regression analysis algorithm was used to identify characteristic genes among the principal targets of MitoDEGs. This analysis used a penalty parameter and 10-fold cross-validation, resulting in the identification of three characteristic MitoDEGs: PRDX4, UCP2, and PHB. Additionally, the SVM-RFE algorithm detected characteristic genes among the key targets of MitoDEGs, employing selected hyperparameters for all classifiers and using 10-fold cross-validation. Four characteristic genes of MitoDEGs were identified: UCP2, DHRS2, PRDX4, and FASN. The final selection of characteristic MitoDEGs, UCP2, and PRDX4 was determined by overlaying results from both LASSO regression analysis and SVM-RFE algorithms using a Venn diagram (Fig. **5C**). Moreover, the expression levels of UCP2 and PRDX4 were validated in the GSE16088 and GSE19276 datasets. A significant decrease in the expression level of UCP2 was observed in OS samples compared to Nor samples, whereas the expression level of PRDX4 exhibited a notable increase in OS samples compared to Nor samples in both the GSE16088 and GSE19276 datasets (Figs. **5D**, **E**). These results suggested that UCP2 and PRDX4 may serve as potential diagnostic biomarkers of OS.

### 3.6 Immune Infiltration and Microenvironments Analysis of Characteristic MitoDEGs

As shown in Figs. (**6A**, **B**), CIBERSORT was used to assess immune cell types in both the GSE16088 and GSE19276 datasets, contrasting OS and Nor samples. Significant differences were observed in the abundance of 15 immune cell types within OS samples, including B cells, macrophages, natural killer (NK) cells, neutrophils, CD4+ T cells, CD8+ T cells, native CD4+ T cells, regulatory T cells (Tr1), natural regulatory T cells (nTregs), Th1 and T helper 2 (Th2) cells, T follicular helper (Tfh) cells, exhausted T cells, native CD8+ T cells, and central memory T cells. Specifically, CD4+ T, CD8+ T, native CD4+ T, Tr1, Th1, Th2, Tfh, exhausted T, and central memory T cells were more abundant in OS samples than in Nor samples, whereas B cells, macrophages, NK cells, neutrophils, nTreg cells, and native CD8+ T cells were more abundant in normal samples. Spearman’s correlation analysis was conducted to explore potential associations between characteristic MitoDEGs and immune cells. In Fig. (**6C**), PRDX4 expression displayed positive correlations with NK cells, effector memory T cells, central memory T cells, CD8+ T cells, Th1 cells, native CD4+ T cells, and infiltration scores, while exhibiting negative associations with B cells and neutrophils (*p* <0.05). Conversely, UCP2 expression positively correlated with neutrophils and B cells, whereas it negatively correlated with native CD4+ T cells, inducible regulatory T cells (iTregs), Th1 cells, cytotoxic T cells, central memory T cells, Tfh cells, Th2 cells, Th17 cells, infiltration scores, nTreg cells, Tr1 cells, monocytes, mucosal-associated invariant T cells, native CD8+ T cells, CD4+ T cells, and dendritic cells (*p* <0.05, *p* <0.01). Additionally, the ESTIMATE algorithm was used to compute the ESTIMATE, immune, stromal, and tumor purity scores for both OS and normal samples. The results indicated that ESTIMATE, immune, stromal, and tumor purity scores were significantly higher in OS samples than in Nor samples (Fig. **6D**).

### 3.7 ScRNA-seq Analysis of Characteristic MitoDEGs and RT-PCR Verification

Using the TISCH database, we explored the expression patterns of characteristic MitoDEGs within the TME of OS at the single-cell level. As shown in Fig. (**7A**), eight distinct cell subsets were identified, including CD4Tconv, CD8Tex, endothelial cells, fibroblasts, malignant cells, mono/macro-phages, osteoblasts, and plasmocytes. Subsequent examination revealed the expression profiles of UCP2 and PRDX4 in these cell subsets (Fig. **7B**). Notably, UCP2 expression was predominantly observed in CD4Tconv, CD8Tex, mono-/macrophages, osteoblasts, and plasmocytes, whereas PRDX4 was primarily expressed in fibroblasts, malignant cells, osteoblasts, and plasmocytes. These findings align with the results obtained from the immune cell infiltration analysis and microenvironment assessments, further corroborating the association of characteristic MitoDEGs with specific cellular populations within the OS TME. Furthermore, RT-PCR confirmed UCP and PRDX4 expression at the cellular level, which was consistent with the bioinformatics results (Fig. **7C**).

## 4 DISCUSSION

OS, the primary malignant bone tumor worldwide, predominantly affects pediatric and young adult populations. Despite advancements in treatment, including surgery and comprehensive treatment modalities, prognostic markers and effective targeted therapies remain limited [24]. Mitochondria are pivotal for cellular metabolism and fate determination, playing crucial roles in cellular bioenergetics, ATP generation to fulfill the energy demands of cells, regulating Ca^2+^ homeostasis and signaling, maintaining redox homeostasis, and determining cell survival or apoptosis [25]. Mitochondria-driven synthesis and catabolic reactions specifically equip OS cells to meet proliferation demands and adapt to adverse survival conditions [26]. Therefore, identifying characteristic MitoDEGs in OS may present a novel avenue for therapeutic targeting and the development of diagnostic biomarkers for this disease. In this study, DEGs were first screened using differential expression analysis between OS and Nor samples in GSE16088 and GSE19276. MitoDEGs were obtained by overlapping the DEGs and mitochondrial genes. LASSO regression analysis and SVM-RFE algorithms were used to select characteristic MitoDEGs and UCP2 and PRDX4 were identified. Given the comprehensive analysis of immune infiltration, the microenvironment, and scRNA-seq, we further analyzed the correlation among obtained characteristic MitoDEGs, immune infiltration, and microenvironments.

UCP2, part of the uncoupling protein family, is predominantly found in tissues that rely on glycolysis. Within the mitochondria, it disrupts oxidative phosphorylation by lowering the mitochondrial membrane potential. The expression level of UCP2 escalates with increasing tumor grades and correlates with a worse prognosis in a population of brain tumor patients, indicating its potential role in patient stratification for metabolic therapies against tumors [27]. This action directly converts energy, resulting in heat dissipation without ATP production (proton leakage) [28]. UCP2 plays a multifaceted role in regulating the development and progression of various conditions. For example, UCP2 can accelerate tumor progression *via* mechanisms that promote aerobic glycolysis (the Warburg effect), inhibit ROS production, and prevent tumor apoptosis, collectively promoting the resistance of cancer cells to chemotherapeutic agents [29]. Conversely, other studies have suggested that UCP2 overexpression may not significantly impact ROS production; however, it could diminish glycolytic activity in cancer cells. In addition, UCP2, through its modulation of ROS generation, influences the inflammatory and immune responses in different pathological conditions, including the adjustment of metabolic requirements in cancer cells and the immune reprogramming of tumors [30].

In this study, we also found that UCP2 was distributed in various immune cells, such as CD4Tconv, CD8Tex, monocytes/macrophages, osteoblasts, and plasmocytes at the single-cell level. Additionally, UCP2 expression positively correlated with neutrophil and B cell infiltration and negatively correlated with native CD8+ T cells, iTregs, Th1 cells, and other immune cell infiltration [31]. Cheng *et al*. proposed that combining UCP2 with immunotherapy, particularly anti-PD1 antibody administration, potentially enhances the antitumor response of CD8+ T cells. Their synergistic approach led to reductions in tumor volume and improvements in survival rates compared to treatment with the anti-PD1 antibody alone [32]. Rupprecht *et al*. [33] proposed that UCP2 plays a role in enhancing immune cell recruitment by directly affecting lymphocytes characterized by elevated UCP2 expression levels. Furthermore, antigenic stimulation of isolated naïve CD4+ and CD8+ T cells induces an increase in UCP2 expression, particularly notable after 24 h and more pronounced upon subsequent restimulation [34]. This suggests that UCP2 acts as a therapeutic biomarker for OS in clinical applications.

PRDX4, a member of the redox protein family, is ubiquitously expressed and distributed across various tissues in mammalian cells [35]. Its primary role is to serve as an endogenous cellular defense mechanism against ROS, particularly in catalyzing the reduction of peroxides to mitigate the accumulation of excessive cellular H_2_O_2_, a byproduct of aerobic metabolism PRDX4 [36]. Given that oxidative stress has been implicated in the activation of inflammation, initiation of tumor transformation, and modulation of tumor progression, considerable attention has been devoted to investigating the role of PRDX4 in tumorigenesis [37]. Increasing evidence has confirmed that high PRDX4 expression is detected in several types of malignant tumors, such as breast cancer, OS, prostate cancer, glioblastoma, and oral cavity squamous cell carcinoma. Hansen *et al*. confirmed the presence of diverse somatic genomic alterations within breast cancer cell lines, which were closely related to the acquisition of docetaxel resistance. Notably, considerable PRDX4 copy number losses on chromosome X were identified, indicating their potential contribution to the emergence of docetaxel resistance. The expression of PRDX4 is elevated in prostate cancer tissues, and the overexpression of PRDX4 notably accelerates the proliferation of prostate cancer cell lines. However, the knockdown of PRDX4 has been observed to diminish proliferation rates variably, contingent upon specific factors [38]. Liu *et al*. suggested that the upregulation of PRDX3 in OS cells may serve as an adaptive response of tumor cells to their microenvironment, facilitating enhanced survival and proliferation while preserving their malignant characteristics and posing potential prognostic significance [39].

Additionally, PRDX4 exhibited high expression levels in fibroblasts, malignant cells, osteoblasts, and plasmocytes at the single-cell level. The expression level was closely related to CD8+ T cells, Th1 cells, and native CD4+ T cells using a comprehensive analysis of immune infiltration, microenvironment, and scRNA-seq, suggesting the pro-oncogenic role of their products in OS. Experimental data have indicated that PRDX4 contributes to the development and progression of immunotherapy [40]. Zhang *et al*. [41] suggested that an increase in PRDX4 expression, coupled with reduced B lymphocyte infiltration and heightened infiltration of CD4+ Th2 T lymphocytes, may impede the antitumor immune response. Conversely, elevations in PRDX4 expression, coupled with augmented infiltration of CD4+-activated memory T lymphocytes and decreased regulatory T cell presence within the TME, could either enhance or diminish antitumor immunity. The impact depends on the degree of influence of gene expression on the immune cells within each subpopulation under different circumstances. Wang *et al*. [42] suggested that NKEF expression increased at the protein level according to iTRAQ results, further confirming the importance of PRDX4 in the immune response.

Additionally, the application of IoT technology in the medical field, particularly in surgery, has made significant strides, revolutionizing the surgical treatment of OS. IoT devices enable remote monitoring and guidance during surgical procedures, enhancing both precision and safety. Furthermore, IoT technology facilitates the collection of patient's physiological parameters and environmental data, which are essential for the bioinformatics analysis of OS [43]. The combined use of machine learning and IoT technology not only improves the efficiency of diagnosing and treating OS but also provides more accurate prognostic assessments, advancing the personalization and precision of OS treatment. As these technologies continue to evolve and become more widely implemented, we anticipate significant improvements in treatment outcomes and quality of life for OS patients.

However, several limitations were noted in our study. The main limitation was the small number of tumor and normal samples. Therefore, applying a larger sample size will help identify diagnostic biomarkers more accurately. Additionally, the mechanism of action of UCP2 and PRDX4 in the progression of OS was not thoroughly investigated in the current study and requires further exploration *via* molecular biology experiments in the future.

## CONCLUSION

We identified UCP2 and PRDX4 as characteristic genes of MitoDEGs, potential diagnostic biomarkers identified *via* machine learning algorithms (LASSO regression analysis and SVM-RFE). We further analyzed their correlation with immune infiltration and the TME at both immune cell and single-cell levels. Furthermore, *in vitro* RT-PCR verification confirmed the expression levels of UCP2 and PRDX4 in OS and osteoblast cells. These findings indicate that UCP2 and PRDX4 may be valuable for evaluating prognosis and facilitating the clinical application of these biomarkers in the diagnosis of OS.

## Figures and Tables

**Fig. (1):**
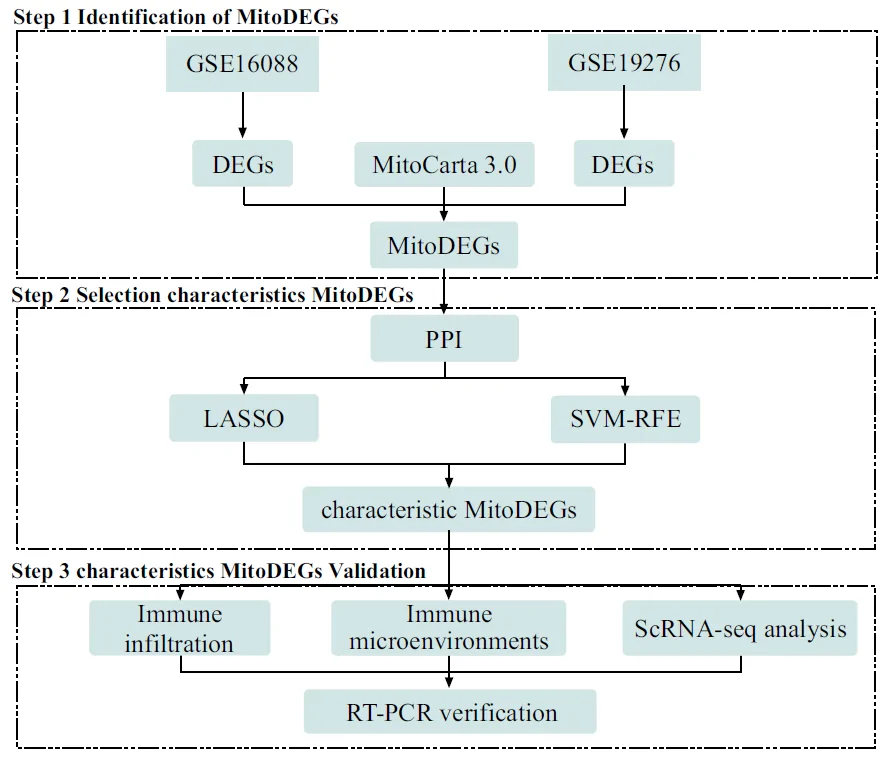
Workflow chart of the analytical process.

**Fig. (2):**
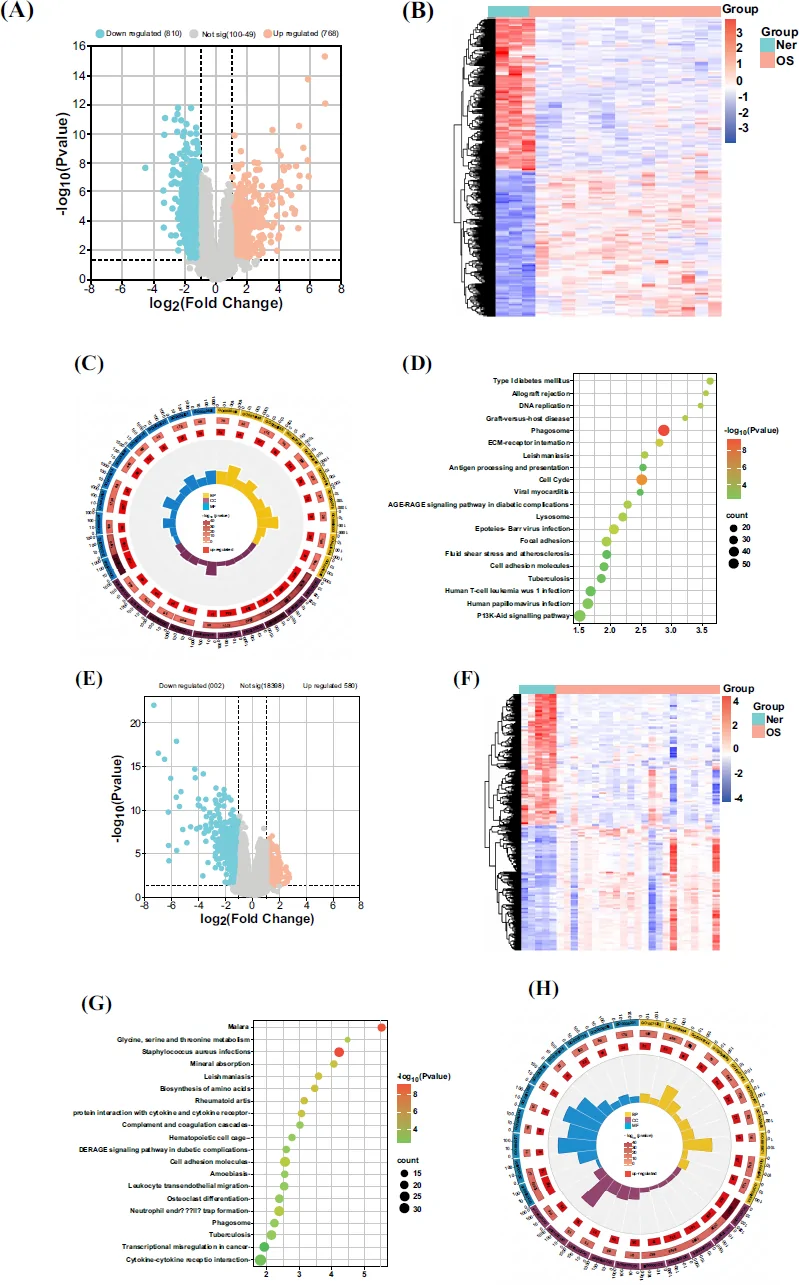
Identification of DEGs in OS datasets and functional enrichment analysis. (**A**) Volcano map of GSE16088, (**B**) Heatmap of DEGs in GSE16088, (**C**) GO analysis of DEGs in GSE16088, (**D**) KEGG pathway analysis of DEGs in GSE16088, (**E**) Volcano map of GSE19276, (**F**) Heatmap of DEGs, (**G**) GO analysis of DEGs in GSE19276 and (**H**) KEGG pathway analysis of DEGs in GSE19276.

**Fig. (3):**
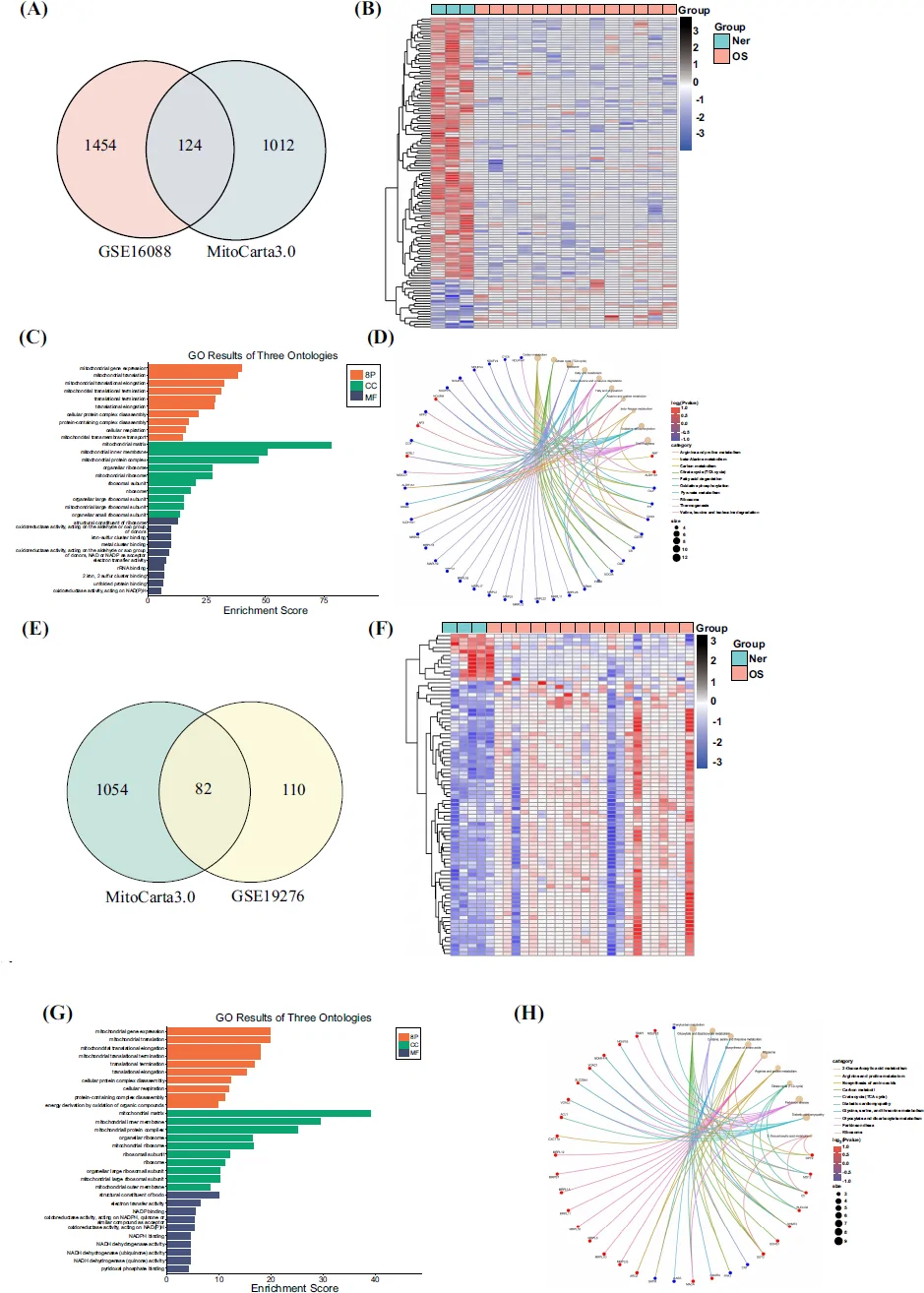
Identification of MitoDEGs in OS datasets and functional enrichment analysis. (**A**) Venn diagrams show the number of DEGs in GSE16088 and MitoCarta 3.0, (**B**) Heatmap of the overlapped MitoDEGs in GSE16088, (**C**) GO analysis of MitoDEGs in GSE16088, (**D**) KEGG pathway of MitoDEGs in GSE16088, (**E**) Venn diagrams show the number of DEGs in GSE19276, (**F**) Heatmap of overlapped MitoDEGs in GSE19276, (**G**) GO analysis of MitoDEGs in GSE19276, and (**H**) KEGG pathway of MitoDEGs in GSE19276.

**Fig. (4):**
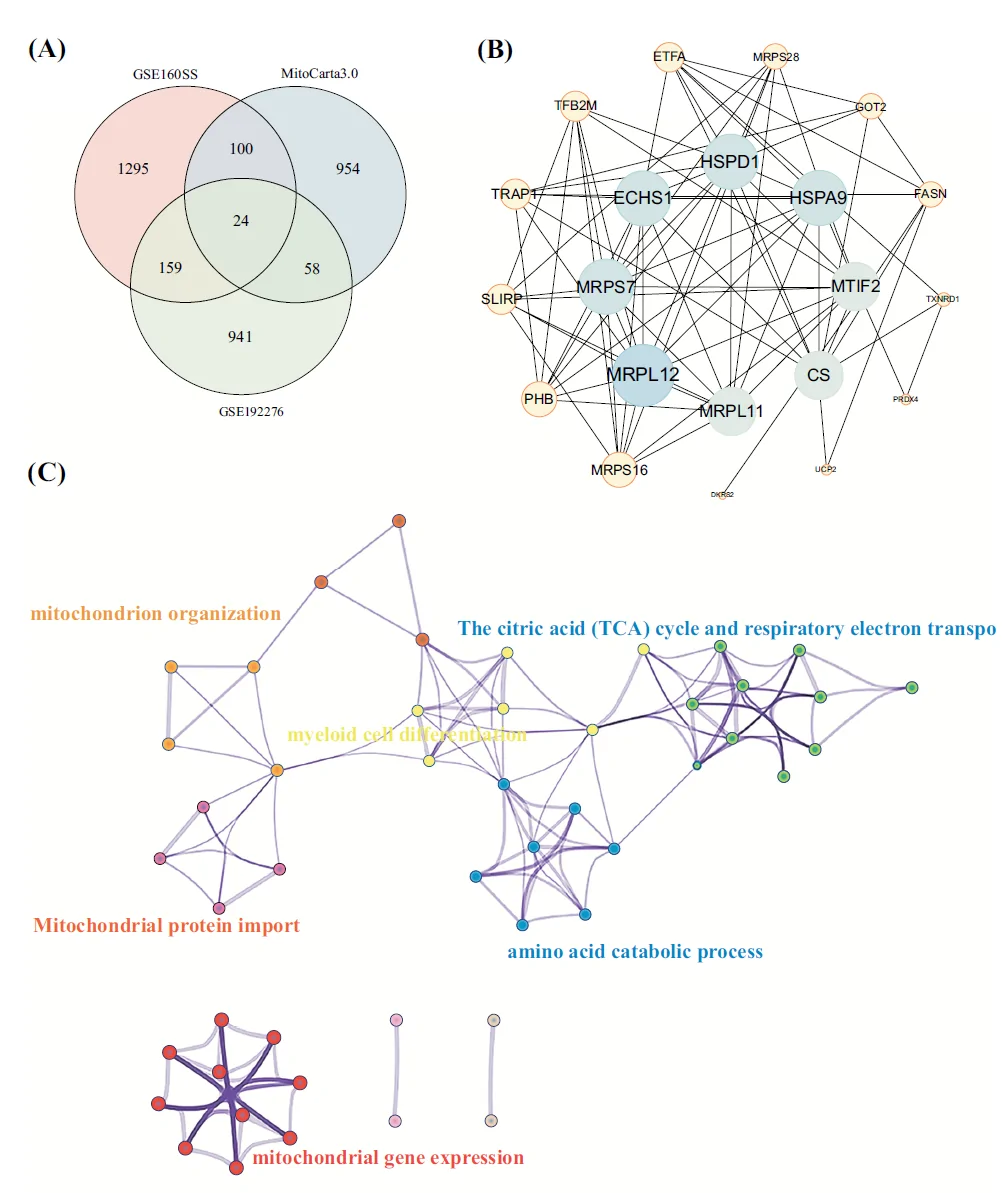
PPI network analysis of MitoDEGs. (**A**) Venn diagram of the DEGs in GSE16088, GSE19276, and MitoCarta 3.0, (**B**) MitoDEG PPI network, and (**C**) GO terms for the MitoDEGs.

**Fig. (5):**
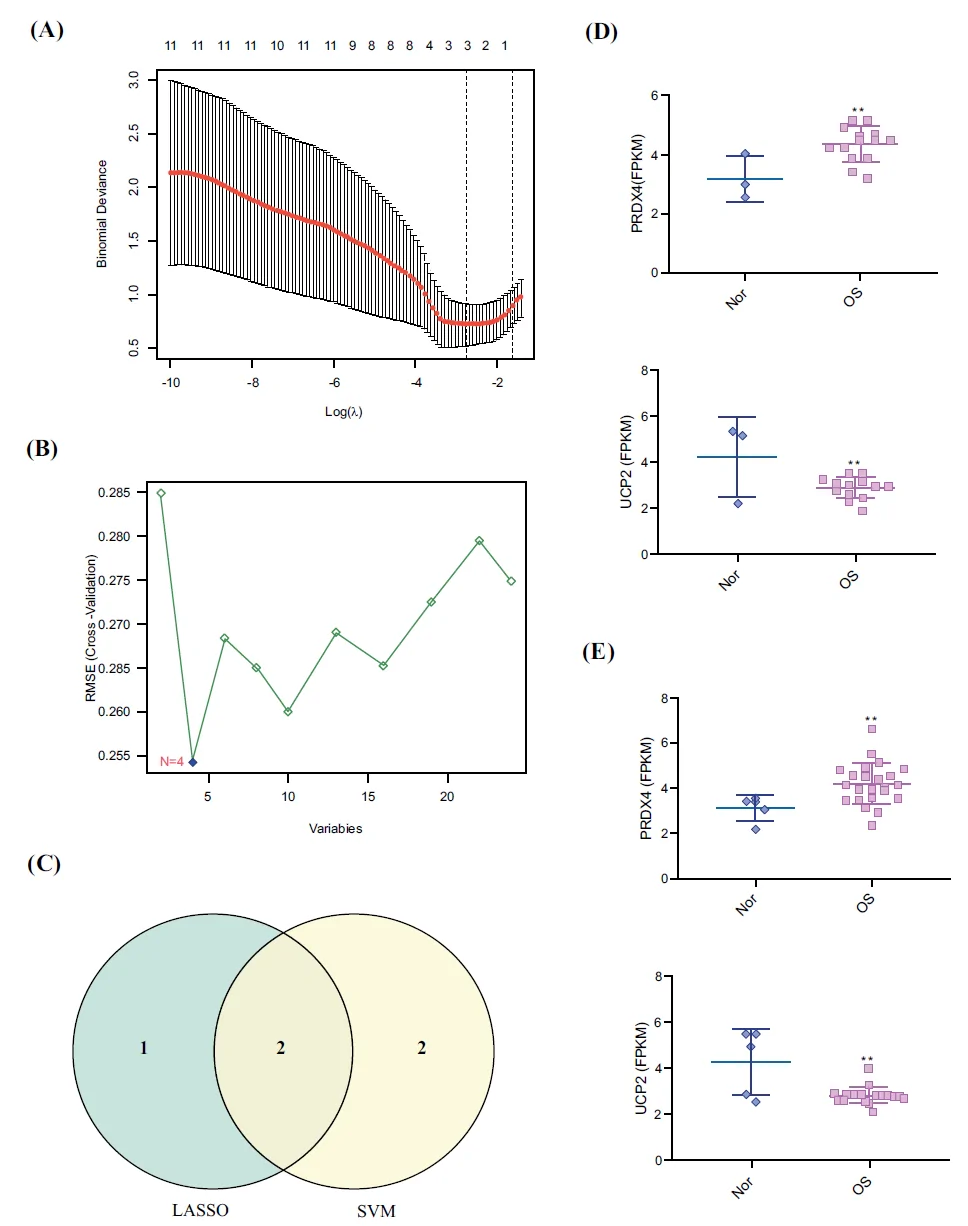
Machine learning screening of the characteristic genes of MitoDEGs. (**A**) LASSO coefficient profiles, (**B**) Estimate generation accuracy for the SVM-RFE algorithm, (**C**) Intersection feature selection between the LASSO and SVM-RFE algorithms, (**D**) Expression levels of PRDX4 and UCP2 in the GSE16088 dataset, and (**E**) Expression levels of PRDX4 and UCP2 in the GSE19276 dataset.

**Fig. (6):**
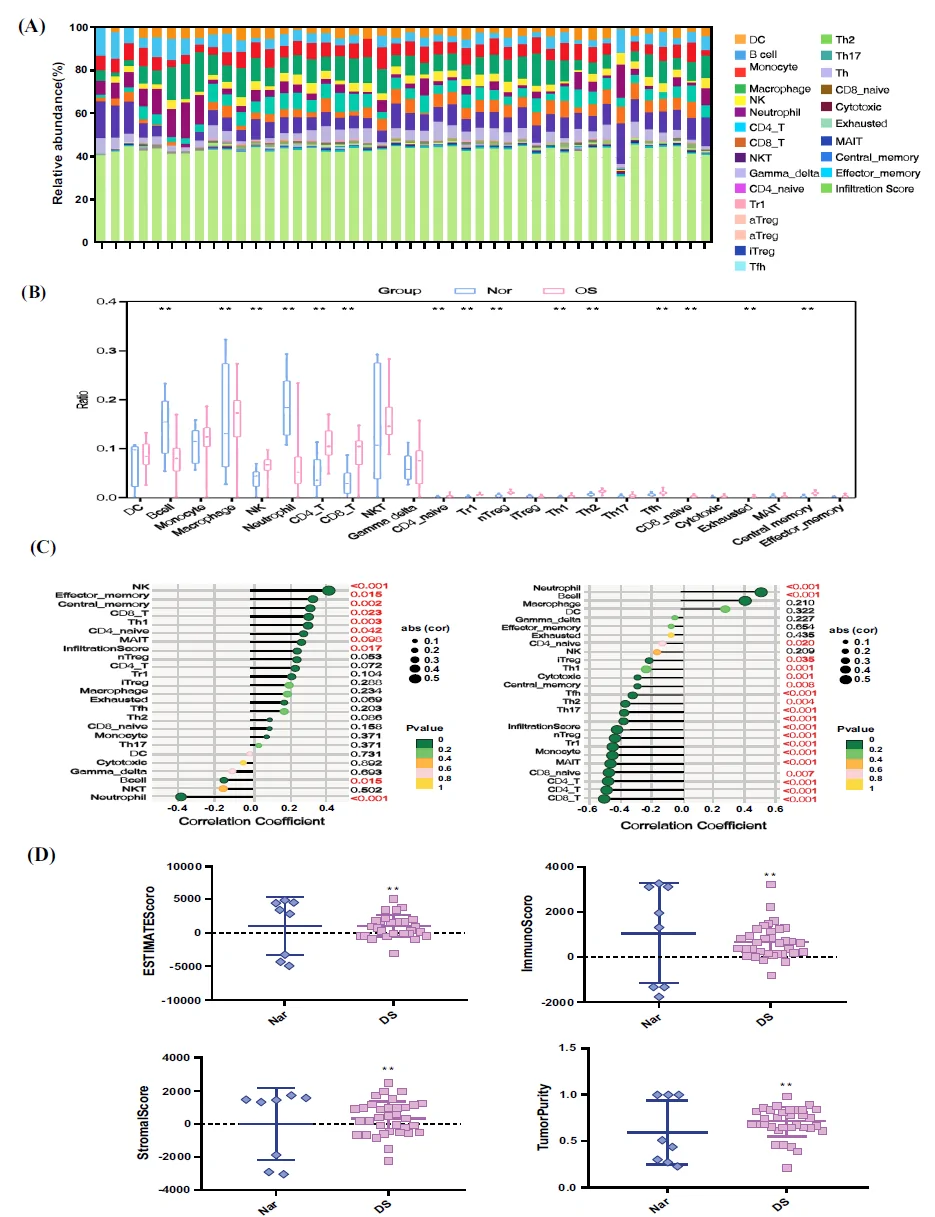
Immune cell infiltration and microenvironment analysis. (**A**) Stacked bar chart of the immune cells, (**B**) Violin plot of immune cell proportions, (**C**) Correlation analysis of PRDX4, UCP2, and immune cells, (**D**) ESTIMATES, Immune, Stromal, and TumorPurity scores analysis (*p <0.05, **p <0.01).

**Fig. (7):**
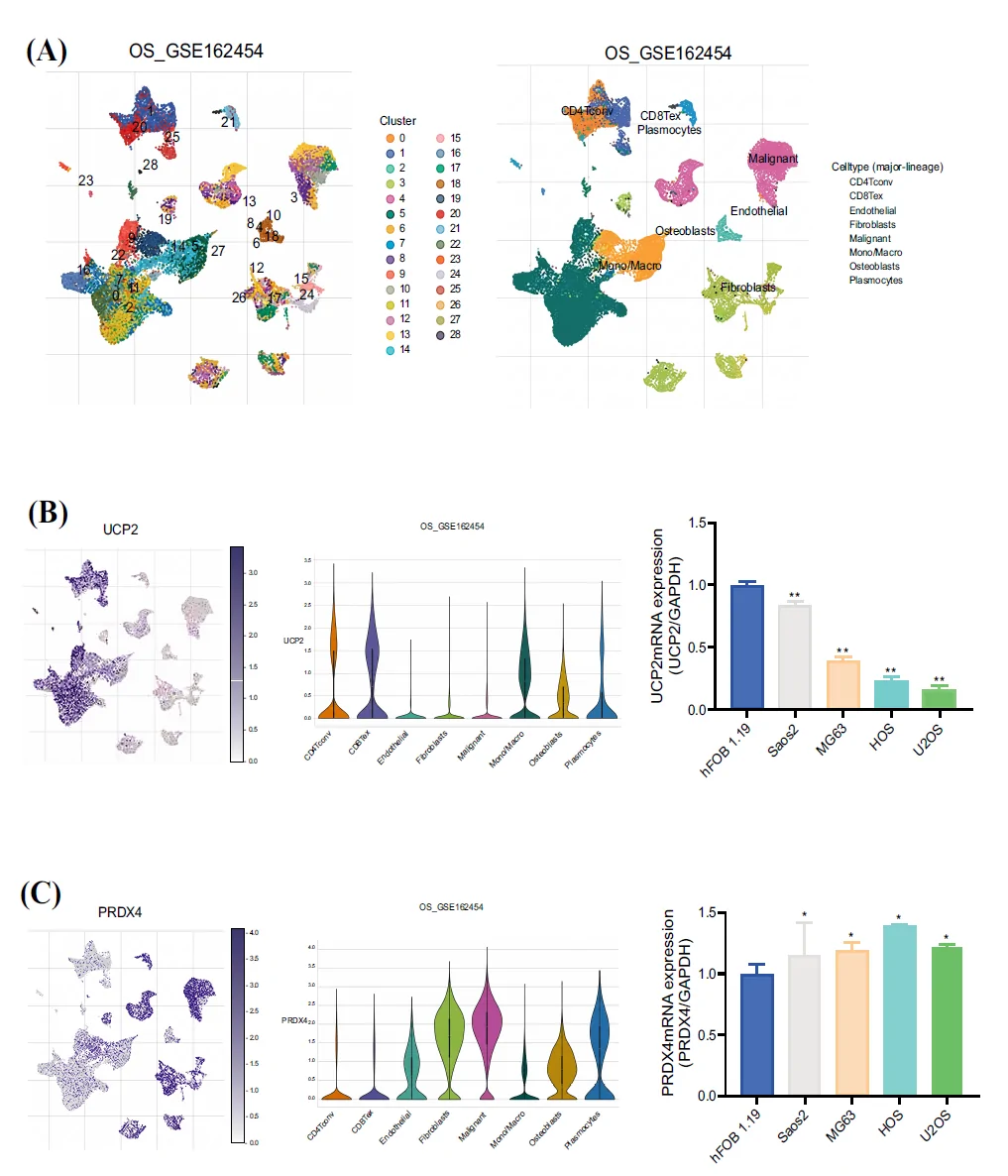
ScRNA-seq analysis of the characteristic genes of MitoDEGs and RT-PCR verification. (**A**) Cell type and distribution in GSE162454; (**B**) Distribution of PRDX4 and UCP2 in GSE162454; (**C**) Expression levels of PRDX4 and UCP2 in four OS cell lines (Saos2, MG63, HOS, and U2OS) and osteoblasts (hFOB 1.19) (*p <0.05, **p<0.01).

## Data Availability

All data generated or analyzed during this study are included in this published article.

## LIST OF ABBREVIATIONS

ANOVA: One-Way Analysis of Variance
ATP: Adenosine Triphosphate
BP: Biological Process
CC: Cellular Component
CT: Computed Tomography
DEG: Differentially Expressed Genes
GEO: Gene Expression Omnibus
GO: Gene Ontology
KEGG: Kyoto Encyclopedia of Genes and Genomes
LASSO: Least Absolute Shrinkage and Selection Operator
MF: Molecular Function
MitoDEGs: Mitochondria-related DEGs
MRGs: Mitochondrial-Related Genes
MRI: Magnetic Resonance Imaging
OS: Osteosarcoma
PPI: Protein-Protein Interaction
PRDX4: Peroxiredoxin 4
ROS: Reactive Oxygen Species
scRNA-seq: Single-Cell RNA Sequencing
SVM- RFE: Support Vector Machines-Recursive Feature Elimination
TCA: Tricarboxylic Acid Cycle
TISCH: Tumor Immune Single-Cell Hub
TME: Tumor Microenvironment
UCP2: Uncoupling Protein 2
